# Development of humanized tri-specific nanobodies with potent neutralization for SARS-CoV-2

**DOI:** 10.1038/s41598-020-74761-y

**Published:** 2020-10-20

**Authors:** Jianbo Dong, Betty Huang, Bo Wang, Allison Titong, Sachith Gallolu Kankanamalage, Zhejun Jia, Meredith Wright, Pannaga Parthasarathy, Yue Liu

**Affiliations:** 1 Ab Studio Inc., Hayward, CA USA; 2 Ab Therapeutics Inc., Hayward, CA USA

**Keywords:** SARS-CoV-2, Antibody therapy

## Abstract

SARS-CoV-2 is a newly emergent coronavirus, which has adversely impacted human health and has led to the COVID-19 pandemic. There is an unmet need to develop therapies against SARS-CoV-2 due to its severity and lack of treatment options. A promising approach to combat COVID-19 is through the neutralization of SARS-CoV-2 by therapeutic antibodies. Previously, we described a strategy to rapidly identify and generate llama nanobodies (VHH) from naïve and synthetic humanized VHH phage libraries that specifically bind the S1 SARS-CoV-2 spike protein, and block the interaction with the human ACE2 receptor. In this study we used computer-aided design to construct multi-specific VHH antibodies fused to human IgG1 Fc domains based on the epitope predictions for leading VHHs. The resulting tri-specific VHH-Fc antibodies show more potent S1 binding, S1/ACE2 blocking, and SARS-CoV-2 pseudovirus neutralization than the bi-specific VHH-Fcs or combination of individual monoclonal VHH-Fcs. Furthermore, protein stability analysis of the VHH-Fcs shows favorable developability features, which enable them to be quickly and successfully developed into therapeutics against COVID-19.

## Introduction

SARS-CoV-2 is a coronavirus that causes the human disease COVID-19, which is contagious and can rapidly spread to cause mild to severe infection, including death [CDC (https://www.cdc.gov/coronavirus/types.html)^1^]. The spread of this newly emergent virus has reached a pandemic level with a significant public impact on the world, leading to more than 25 million infections and more than a 0.85 million deaths worldwide [World Health Organization (WHO) (https://www.who.int/emergencies/diseases/novel-coronavirus-2019)]. In addition to threatening human health, COVID-19 has also caused a significant socio-economic impact around the world [United Nations (https://www.undp.org/content/undp/en/home/coronavirus/socio-economic-impact-of-covid-19.html)].

Although there are relatively successful diagnostic methods to detect the SARS-CoV-2 infection in humans, there are currently no successful therapies that can interfere with virus replication. The small antiviral molecule Remdesivir (Gilead) which inhibits the RNA-dependent RNA polymerase of SARS-CoV-2 decreases the recovery time in patients with COVID-19^2^, but it most likely cannot completely stop or prevent SARS-CoV-2 infections in humans. Another small antiviral molecule, GRL-0617, shows promise in interfering with the SARS-CoV-2 replication by inhibiting the papain-like protease, however, it is yet to be tested in clinical trials^3^. Moreover, there are no FDA-approved vaccines to prevent SARS-CoV-2 infections in humans, although several groups are currently in the pursuit such vaccines [WHO (https://www.who.int/publications/m/item/draft-landscape-of-covid-19-candidate-vaccines)]. Therefore, rapid development of therapeutics and preventative strategies has become an essential and urgent need to fight the COVID-19 pandemic.

The trimeric spike (S) proteins that protrude through the envelope of the SARS-CoV-2 virion mediate virus entry into the host cells by interacting with the human ACE2 receptor^4–9^. Therefore, a major target for anti-SARS-CoV-2 neutralizing antibodies in development are to block the interaction of SARS-CoV-2 S1 protein with ACE2. In particular, two popular strategies have been employed to discover and develop monoclonal IgG antibodies that can recognize SARS-CoV-2 S1 protein mainly by binding to its receptor binding domain (RBD)^10–15^. The first commonly used method is to clone the antibody V genes from the B cells of surviving COVID-19 patients who have mounted a natural immune response against SARS-CoV-2^10,11,13^. This strategy has yielded a number of neutralizing monoclonal antibodies; however, it is important to note that the patients’ antibody repertoire condition and the timing of blood sample collection play a critical role in its success. The other well-recognized and classic approach for antibody generation is by immunizing humanized mice^15^. Additionally, new SARS-CoV-2 antibodies were developed by screening cross-neutralizing antibodies for the SARS-CoV-2 S1 protein binders from the antibodies that were initially tested or developed to treat SARS by blocking SARS-CoV S/ACE2 or MERS by blocking MERS-CoV S/CD26 interactions^12,14^. One of the cross-binders is a single domain antibody/nanobody (VHH) generated from SARS-CoV S-immunized llama^14^. Moreover, VHHs against SARS-CoV-2 have also been generated from the llama VHH libraries^16^. The approach of using camelid antibody VHHs is advantageous because the VHH regions are easy to produce, are stable, and are smaller sized, which increases the possibility to target unique epitopes that are not accessible to conventional VH/VL antibodies^17,18^.

In our recently published and follow-up studies, we identified more than 80 VHH binders against SARS-CoV-2 S1 protein from naïve and synthetic humanized llama VHH libraries^19^, out of which 19 had S/ACE2 blocking ability. Then, we analyzed the synergistic effects of combining pairs of S/ACE2 blocking candidates, which led to the construction of bi-specific VHH-Fc antibodies which are significantly more potent than the individual monoclonal VHH-Fcs in SARS-CoV-2 S1 RBD binding and S/ACE2 blocking^19^. Based on our findings with the bi-specific VHH-Fc and computer-aided epitope modeling predictions, we reasoned that adding a third VHH would further increase the synergistic potency in tri-specific antibodies. In this manner, we designed and constructed several tri-specific VHH-Fc antibodies with enhanced efficacy compared to previously generated bi-specific antibodies as well as using three monoclonal antibodies in combination, confirming our rational. These tri-specific VHH-Fcs are not only extremely potent in SARS-CoV-2 S1 RBD binding and S/ACE2 blocking, but also potently inhibited the infection of human target cells by a SARS-CoV-2 pseudovirus. Moreover, our protein stability assessments show that the lead tri-specific VHH-Fc antibodies have favorable developability features for large-scale manufacturing. Together, these results indicate that the tri-specific VHH-Fc antibodies are promising therapeutics in COVID-19 treatment and prevention.

## Results

### Identification of VHHs binding to different epitopes of SARS-CoV-2 S1 protein RBD

**Figure 1:**
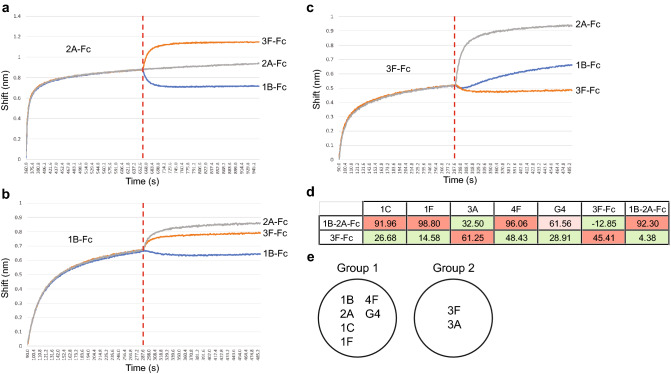
Identification of VHHs binding to different epitopes of SARS-CoV-2 S1 protein RBD. Epitope binning of VHH-Fcs were assessed on the Gator (Probe Life) using (**a**) 2A-Fc-loaded, (**b**) 1B-Fc-loaded, and (**c**) 3F-Fc-loaded RBD sensors which quantify the wavelength shift (indicative of binding signal) over time. (**d**) Epitope binning of VHHs were assessed using an ELISA method. Briefly, the SARS-CoV-2 S1 protein was incubated with 1B-2A-Fc or 3F-Fc, and binding competition was performed with the VHHs followed by the detection of the biotinylation. The experiment was performed in duplicates, and the average percent difference from the competing pairs relative to the VHH-Fc alone signal are indicated in the table. The VHH associated percentages highlighted in Red are likely high VHH competitors, in Light Red are partial competitors, and in Green are likely non-competitors. (**e**) The two Groups of VHHs categorized based on the binding to epitopes on S1 RBD.

Recently, we reported the identification of llama VHHs that bind to the SARS-CoV-2 S1 protein RBD^19^. Briefly, we used two llama VHH libraries (one naïve library and another humanized synthetic library derived from the naïve library) to screen for VHHs that bind to the SARS-CoV-2 S1 protein in-vitro^19^. We identified a total of 89 S1 protein binders, 64 from the naïve and 25 from the humanized libraries. Out of the S1 protein binders, 19 VHHs blocked the interaction between SARS-CoV-2 S1 RBD and ACE2 receptor, with 12 S/ACE2 blockers identified from the naïve library and 7 identified from the humanized library (data not shown). Furthermore, we observed that the pairwise addition of some of the VHHs caused synergistic effects on SARS-CoV-2 S/ACE2 blocking^19^. We hypothesized that this synergistic effect is caused by binding of the VHHs to different epitopes within the S1 RBD. To test this, we performed epitope binning assays by biolayer interferometry (Fig. 1a–c) or ELISA (Fig. 1d) on a selected number of candidates.

In the initial epitope binning assay (Fig. 1a–c), we used an S1 RBD sensor to capture 2A-Fc, 1B-Fc, or 3F-Fc separately, followed by the incubation with our lead candidates 1B-Fc, 3F-Fc or 2A-Fc. The VHHs were fused to human IgG1 Fc domains to render the Fc effector functions against SARS-CoV-2^19^. This analysis showed that with the 2A-Fc-loaded probe, the addition of 3F-Fc further increased the signal compared to the 2A-Fc control, while the addition of 1B-Fc decreased the signal compared to the control (Fig. 1a). This indicates that 3F-Fc does not compete with the 2A-Fc site and it is likely that they bind to different S1 RBD epitopes. In contrast, 1B-Fc competed with 2A-Fc, indicating that they compete for binding to the same S1 RBD epitope (Fig. 1a). Similarly, with the 1B-Fc-loaded probe (Fig. 1b), 3F-Fc increased the signal compared to the 1B-Fc control. This shows that 3F-Fc does not compete with 1B-Fc. Interestingly, 2A-Fc also increased its signal compared to 1B-Fc control. This suggests that although having a common binding region, 2A binds to a wider epitope than 1B (Fig. 1b). With 3F-Fc-loaded probe, both 2A-Fc and 1B-Fc showed an increase of the signal compared to the 3F-Fc control. This further shows that 3F-Fc does not compete with either 1B-Fc or 2A-Fc, and likely bind to a different epitope (Fig. 1c). These results confirm our hypothesis and show that S/ACE2 blocking VHHs bind to at least two separate unique epitopes within the S1 RBD.

Next, we performed an ELISA-based epitope binning assay to assess five additional VHHs (1C, 1F, 3A, 4F, and G4) unfused to Fc, but previously assessed to block the SARS-CoV-2 S/ACE2 interaction^19^. The assessment of more VHHs would allow us to categorize several of our other VHHs into binding groups, which will aid in multi-specific antibody design and construction. In this ELISA, wells were coated with SARS-CoV-2 S1 and incubated with bi-specific VHH-Fc 1B-2A (based on previous data, 1B and 2A likely bind the same epitope) or monoclonal VHH-Fc 3F-Fc (based on previous data, this binds a different epitope than 1B or 2A) premixed with the VHH candidates. The resulting relative fluorescence signals obtained for each sample were calculated to reflect the percent difference from 1C, 1F, 3A, 4F, G4, and controls (3F-Fc and 1B-2A-Fc) signals, when the VHHs are combined with 1B-2A-Fc or 3F-Fc (Fig. 1d). The results show that VHH-Fcs 1C, 1F, 4F, as well as the 1B-2A-Fc control have almost 100% difference from 1B-2A-Fc, which highly suggest that they compete for the same epitope (Highlighted in Red). However, G4 (Highlighted in Light Red) may partially compete with 1B-2A-Fc, whereas 3A does not likely compete for the same epitope (Highlighted in Green). Additionally, these results show that 3A and the 3F-Fc control may compete with 3F-Fc (Fig. 1d), while other VHHs, including the 1B-2A-Fc control resulted in a lower percent difference. We also performed additional epitope binning assays using biolayer interferometry to assess the competition of the VHH-Fcs 1C, G4, and 3A to bind to S1 RBD. The VHH-Fcs 1F and 4F poorly bound to the biolayer interferometry probes used for this assay and were excluded from analysis. This approach confirmed the results that we obtained by ELISA and showed that 1C and G4 likely belong to Group 1, and 3A belongs to Group 2 in terms of the binding competition (Supplementary Fig. 1). Interestingly, G4-Fc shows competition with either 1B-Fc and 2A-Fc when it is loaded onto the probe first (Data not shown). In contrast, reversal the of loading further increased its signal compared to both 1B-Fc control and 2A-Fc control, suggesting that the epitope for G4 is wider than that of both 1B and 2A (Supplementary Fig. 1). Taken together, we could categorize 8 VHH blockers of S/ACE2 interaction into 2 major groups based on their epitope binding; Group 1 consist of 6 VHHs, whereas Group 2 consist of 2 VHHs (Fig. 1e).

### Elucidation of epitopes on S1 RBD that bind to VHH-Fcs

**Figure 2:**
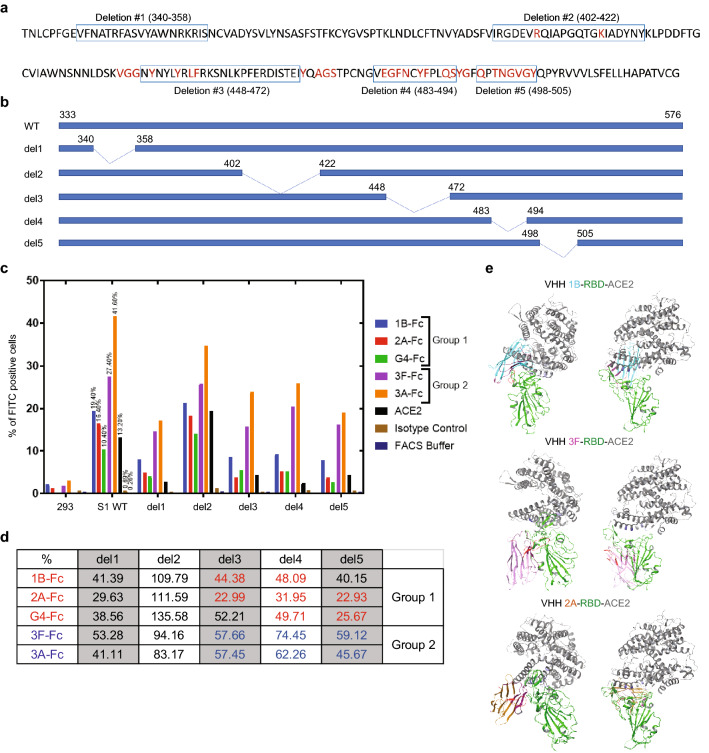
Elucidation of epitopes on S1 RBD that bind to VHH-Fcs. (**a**) ACE2 binding residues on SARS-CoV-2 S1 RBD were determined by Schrodinger BioLuminate based on the protein–protein interactions of Protein Data Bank (PDB) 6M0J. (Schrödinger Release 2020-3: BioLuminate, Schrödinger, LLC, New York, NY, 2020. https://www.schrodinger.com/products/bioluminate. Requires permission to be used). The residues in Red are predicted ACE2 interactors. The deletions generated on SARS-CoV-2 S1 RBD are shown with the boxed regions. (**b**) Deletion map schematics of the S1 RBD deletion mutants. (**c**) The binding of VHH-Fcs and ACE2 to Expi293 cells expressing SARS-CoV-2 S1 wild type (WT) or mutant proteins (del1–del5) were assessed by flow cytometry following FITC-conjugated secondary antibody treatment. An isotype control antibody and FACS buffer were used as negative controls. The experiment was performed at least three times which yielded similar trends in results. A representative image of a single experiment is shown here. The graph was generated by the Prism (GraphPad) software (Prism version 8.4.3. https://www.graphpad.com/scientific-software/prism/. Requires permission to be used) (**d**) In the experiment shown in (**c**), the binding percentages relative to the S1 WT for each VHH-Fc were calculated in the context of each deletion mutant. The Group 1 VHH-Fcs and the values with binding differences that contributed to their categorization into that group are shown in Red. Those values for Group 2 VHH-Fcs are shown in Blue. (**e**) Docking models between SARS-CoV-2 S1 RBD and the lead VHHs generated with Schrodinger BioLuminate software. (Schrödinger Release 2020-3: BioLuminate, Schrödinger, LLC, New York, NY, 2020. https://www.schrodinger.com/products/bioluminate. Requires permission to be used).

In an effort to elucidate the structural basis of the newly discovered epitope binding groups, we computationally generated structural models for 1B, 3F, and 2A VHHs and docked them with SARS-CoV-2 S1 RBD structure exported from PDB 6M0J using Schrodinger BioLuminate software^20–22^. For context, Fig. 2a shows the SARS-CoV-2 S1 protein with the ACE2 binding residues in red font. This approach generated an array of poses of each S1 RBD/VHH complex structure, which allowed us to further analyze the interfaces of those poses with a good PIPER cluster size and led us to identify five regions in the RBD which may interact with VHH 1B, 2A, and 3F, respectively (Fig. 2a,b)^23–25^. Next, we generated 5 different S1 RBD deletion mutants to validate the computationally mapped epitopes in-vitro to select the best docking model for molecular analysis. Interestingly, these S1 RBD deletion regions have been shown to mediate the S1 RBD/ACE2 interaction in recently published literature^10–13,26^ (Table 1). We tested wild-type and all the S1 deletion mutants for their ability to bind to a tri-specific VHH-Fc to check whether the proteins are folded and expressed on the cells. The results show that they are indeed expressed and folded as they all bind to the tri-specific VHH-Fc, although the level of expression and/or folding might be different across the mutants based on the strength of the binding signals. The wild-type S1 RBD and the deletion 2 (del2) shows stronger binding, whereas the deletions 1 (del1), 3 (del3), 4 (del4) and 5 (del5) show weaker binding to the tri-specific VHH-Fc (Supplementary Fig. 2). Then we assessed the binding profiles of the S1 RBD wild-type and the deletion mutants with selected VHH-Fcs from Group 1 and Group 2, as well as ACE2 (Fig. 2c,d). The binding of VHH-Fc candidates from both Group 1 and Group 2, as well as ACE2 to S1 RBD are affected following the removal of del1. It is possible that this result is due to a conformational change or decrease of S1 protein expression following its deletion because based on crystal structure of the RBD/ACE2 complex (PDB 6M0J), the deleted domain is not part of the S1 RBD/ACE2 interface. The del2 mutant, which is adjacent to a computationally-predicted epitope domain in region 1, does not prevent the binding of both Group 1 and Group 2 VHH-Fcs to S1 RBD. In addition, it does not prevent the binding of ACE2 to S1 RBD. The del3, 4, and 5 mutants all decreased binding of both Group 1 and Group 2 VHH-Fcs to S1 RBD. However, these regions are more critical for Group 1 than Group 2 for their binding. In addition, these regions are critical for ACE2 to bind to S1 RBD. Taken together, the binding epitopes for Group 1 is more associated with del3, 4 and 5 regions which are located at the interface of S1 RBD/ACE2, while at least part of the epitopes for Group 2 are shifted farther away from the S1 RBD/ACE2 interface relative to the epitopes for Group 1 VHHs (Fig. 2c,d). Based on the binding and epitope binning data, we constructed 3D docking models that predicted the interactions between SARS-CoV-2 S1 RBD, ACE2 and lead VHH-Fcs (Fig. 2e). These models show that predicted binding epitopes for Group 1 VHHs 1B and 2A are located at the S1 RBD/ACE2 interface. In contrast, the epitope for Group 2 VHH 3F is located away from the S1 RBD/ACE2 interface (Fig. 2e). Interestingly, there are binding variations seen within Group 1. The binding of 2A to del1, del3, del4 and del5 have decreased more than that of 1B. This shows that epitopes for 2A and 1B are not the same even though they compete with each other and were initially characterized to be within the same binding Group 1 (Fig. 2c,d). Taken together, our analysis confirms that there are two major binding groups (Group 1 and Group 2) and we show the likely binding regions on the SARS-CoV-2 S1 protein for each VHH.

**Table 1:** List of S1 RBD deletions and published antibodies that target the deleted regions for S/ACE2 interaction blocking.

SARS-CoV-2 S1 RBD deletions	Published antibodies targeting the deleted region
S1 RBD del 1	S309^12^, BD-23^13^
S1 RBD del 2	CB6^10^
S1 RBD del 3	B38^26^, CB6^10^, P2B-2F6^11^
S1 RBD del 4	B38^26^, CB6^10^, P2B-2F6^11^
S1 RBD del 5	B38^26^, CB6^10^

### Tri-specific VHH-Fcs show potent S1 RBD binding and S/ACE2 blocking activity

**Figure 3:**
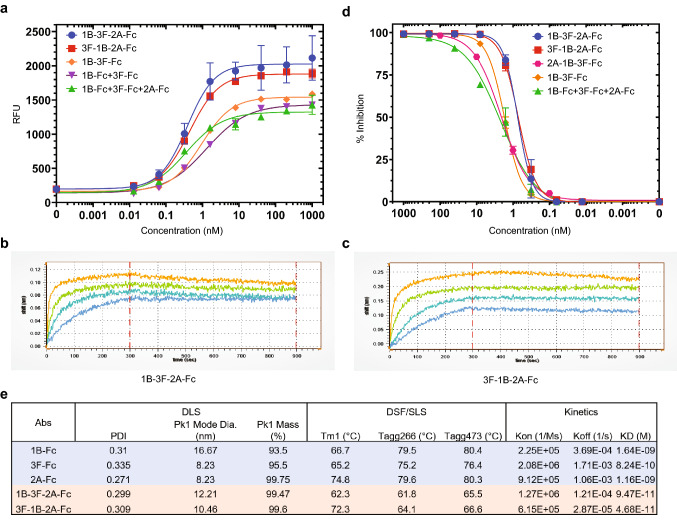
Tri-specific VHH-Fcs show potent S1 RBD binding and S/ACE2 blocking activity, and favorable developability features. (**a**) Binding of multi-specific and monoclonal VHH-Fcs to SARS-CoV-2 S1 protein at different concentrations was assessed in duplicates using an ELISA method. The binding signal is based on fluorescence, indicated as Relative Fluorescence Units (RFU). Error bars represent standard deviation. The graph was generated by the Prism (GraphPad) software (Prism version 8.4.3. https://www.graphpad.com/scientific-software/prism/. Requires permission to be used). (**b**, **c**) Binding kinetic graphs for tri-specific VHH-Fcs were obtained by biolayer interferometry (Gator). B and C represent the graphs for 1B-3F-2A-Fc and 3F-1B-2A-Fc, respectively. (**d**) Blocking of SARS-CoV-2 S/ACE2 interaction by multi-specific and monoclonal VHH-Fcs at different concentrations was assessed in duplicates using an ELISA method. Percent inhibition was calculated based on the blocking signal in RFU for each VHH-Fc treatment. Error bars represent standard deviation. The graph was generated by the Prism (GraphPad) software (Prism version 8.4.3. https://www.graphpad.com/scientific-software/prism/. Requires permission to be used). (**e**) Developability features examining the biophysical and chemical characteristics of VHH-Fcs using DLS (Dynamic light scattering), DSF (Differential scanning fluorimetry), SLS (Static light scattering). The kinetic values were obtained by biolayer interferometry (Gator).

Next, we tested whether the combination of individual VHHs binding to different S1 RBD epitopes into bi-specific antibody molecules would yield synergistic effects in SARS-CoV-2 binding and S/ACE2 blocking. As expected, the resulting bi-specific VHH-Fc 1B-3F showed superior binding to S1 RBD and S/ACE2 blocking compared to individual component VHH-Fcs^19^. Since SARS-CoV-2 S proteins formed trimers, we started to study whether tri-specific antibodies with two binding units from Group 1 and another binding unit from Group 2 or vice versa would have better binding and blocking function than the bi-specific antibody^27–29^. Here, we only focused on tri-specific, as any larger multi-specific molecules will likely affect developability with Fc fusion proteins. We selected the VHHs from both Group 1 and 2 with the most favorable binding, functional and developability features, and constructed tri-specific VHH-Fcs with the computer-aided antibody design using the software BioLuminate (Schrodinger) that enabled their effective construction and optimization^20–22^. Then, we tested the tri-specific, bi-specific and mono-specific VHH-Fcs for their ability in-vitro for SARS-CoV-2 S1 protein binding and S/ACE2 blocking (Fig. 3a,d). As expected, the multi-specific antibodies showed higher binding affinities to SARS-CoV-2 S1 protein RBD in-vitro, with the tri-specific VHH-Fcs 3F-1B-2A (KD ~ 0.047 nM) and 1B-3F-2A (KD ~ 0.095 nM) showing more potent binding than bi-specific VHH-Fc 1B-3F (Fig. 3a–c,e). The binding affinities for tri-specific VHH-Fcs were higher than that of individual component VHH-Fcs 1B, 3F and 2A used in combination, and the binding affinity for 1B-3F-Fc was higher than that of individual component VHH-Fcs 1B, and 3F used in combination (Fig. 3a). In addition, 3F-1B-2A and 1B-3F-2A showed potent blocking of the SARS-CoV-2 S/ACE2 interaction, with IC_50_ values of 0.71 nM and 0.74 nM, and full inhibition around 10 nM for both, respectively, that were far superior to using individual component VHH-Fcs as combinations (IC_50_ of 2.21 nM and full inhibition around 100 nM). In addition, 3F-1B-2A and 1B-3F-2A were more potent than bi-specific VHH-Fc 1B-3F in blocking SARS-CoV-2 S/ACE2 interaction (Fig. 3d). Interestingly, the tri-specific VHH-Fc 2A-1B-3F had lower S/ACE2 blocking ability showing the physical arrangement and/or binding orientation of the VHHs in a multi-specific antibody is important for its binding and blocking (Fig. 3d). Taken together, this data indicates that the tri-specific VHH-Fcs have a higher synergistic potency in both binding and blocking the S1 or S1/ACE2 interaction than bi-specific or mono-specific antibodies.

### Tri-specific VHH-Fcs have favorable developability features

During the computer-aided design process, we incorporated several development-enhancing features in the structures of our VHH-Fcs. Therefore, we analyzed the physico-chemical properties, using DLS and DSF/SLS methods, of our lead bi- and tri-specific antibodies to determine whether they possess favorable characteristics for large-scale manufacturing that is essential for the commercial development of the antibodies (Fig. 3e). Our data revealed that the lead tri-specific VHH-Fc 3F-1B-2A has lower aggregation potential based on the DLS method and is thermostable based on the DSF/SLS method (Fig. 3e).

### Tri-specific VHH-Fc 3F-1B-2A neutralizes SARS-CoV-2 infection in cells

**Figure 4:**
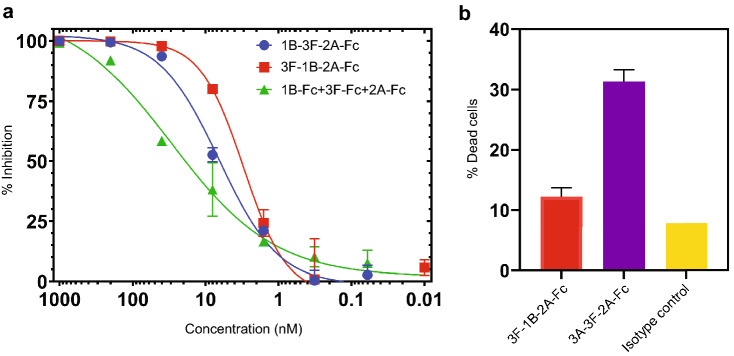
Tri-specific VHH-Fc 3F-1B-2A neutralizes SARS-CoV-2 infection in cells. (**a**) Blocking of SARS-CoV-2 pseudovirus infection by VHH-Fc 3F-1B-2A. HEK293 ACE2/TMPRSS2 cells were incubated with SARS-CoV-2 pseudovirus and treated with 1:5 serial dilutions of VHH-Fcs, starting at 1000 nM in triplicates. Percent inhibition was calculated based the luminescence signal in RLU for each VHH-Fc treatment. Error bars represent standard error of the mean. (**b**) ADCC function was assessed for the tri-specific VHH-Fcs (3F-1B-2A, 3A-3F-2A and an isotype control antibody) in duplicates. Cell death percentage was calculated based on cell percentage of VHH-Fc treated cells in comparison to the isotype control. Error bars represent standard deviation. The graphs in both (**a**) and (**b**) were generated by the Prism (GraphPad) software (Prism version 8.4.3. https://www.graphpad.com/scientific-software/prism/. Requires permission to be used).

We tested the multi-specific VHH-Fcs for their ability to target SARS-CoV-2 in cell biological functional assays. First, we analyzed the virus neutralizing ability of our antibodies using a pseudovirus that expresses the SARS-CoV-2 S1 protein. The tri-specific VHH-Fcs 3F-1B-2A, 1B-3F-2A, and the mono-specific combinations of VHHs (1B-Fc + 3F-Fc + 2A-Fc) prevented the infection of human cells by the pseudoviruses (Fig. 4a). In accordance with the SARS-CoV-2 S/ACE2 blocking data, the tri-specific VHH-Fcs were more effective in neutralizing the pseudovirus infection than the combination treatment of VHH-Fcs 1B, 3F and 2A, with IC_50_ values of 3.00 nM for 3F-1B-2A, 6.44 nM for 1B-3F-2A, and 29.19 nM for the combination treatment. (Fig. 4a). This pseudovirus data presented here confirm the synergistic effect of the tri-specific antibodies and most importantly, it suggests that it is likely effective in preventing the SARS-CoV-2 infection.

As our VHH-Fcs contain the Fc domain of human IgG1, we expected it would be able to trigger the Fc-dependent functions to eliminate the viruses from the body. To test this, we used a cell line that transiently expresses the SARS-CoV-2 S1 protein. Then, we assessed the ability of our multi-specific VHH-Fcs to promote antibody‐dependent cellular cytotoxicity (ADCC) that is an Fc-dependent function of the antibodies. In addition to our lead tri-specific VHH-Fc antibody 3F-1B-2A, we also tested 3A-3F-2A-Fc, another tri-specific antibody we constructed with similar S1 binding and S/ACE2 blocking potency (Supplementary Fig. 3). As expected, the VHH-Fcs were able to induce ADCC in the cells (Fig. 4b). This suggests that these VHH-Fcs could bind to immune cells through their Fc domain and elicit Fc-dependent functions, thereby allowing multiple mechanisms of actions against SARS-CoV-2, including binding SARS-CoV-S1 and blocking S1/ACE2 interactions.

### Generation of a structure docking model showing the interaction of 3F-1B-2A-Fc with SARS-CoV-2 S1 RBD

**Figure 5:**
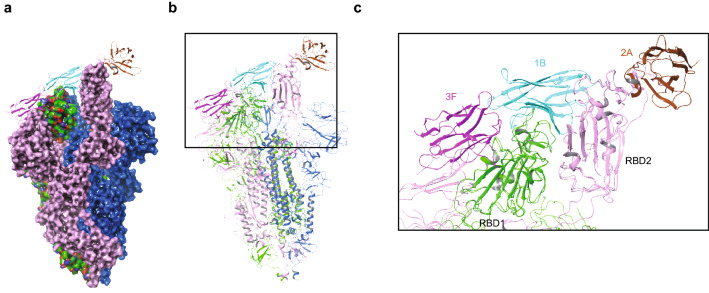
Structure docking model showing how 3F-1B-2A-Fc interacts with SARS-CoV-2 S1 RBD. 3D docking model for SARS-CoV-2 S1 RBD with tri-specific VHH-Fc 3F-1B-2A was generated by BioLuminate (Schrödinger Release 2020-3: BioLuminate, Schrödinger, LLC, New York, NY, 2020. https://www.schrodinger.com/products/bioluminate. Requires permission to be used). In the software, the SARS-CoV-2 RBD spike protein trimer (PDB 6X2A) was split into three monomeric forms (Chain A, B and C). Then, 1B/3F/RBD model structure was aligned with chain A of PDB 6X2A to create Group 1 and 2A/RBD model structure was aligned with chain B of PDB 6X2A to create Group 2. Then, Group 1, Group 2 and chain C were merged to generate the final structure. The S1 RBD/VHH docking structure is represented with a surface structure (**a**) and ribbon structure (**b**). The enlarged S1 RBD/VHH docking structure is shown in right (**c**).

Using a computational approach, we generated a 3-dimensional docking model depicting the interaction of our lead tri-specific antibody 3F-1B-2A-Fc with the RBDs of SARS-CoV-2 S1 with BioLuminate software^20–22^. This model predicted how the individual VHH-Fcs, belonging from two binding groups, can interact with a single RBD using different epitopes. In addition, this model suggests that individual VHHs of the tri-specific VHH-Fc interact with multiple RBDs in the SARS-CoV-2 S trimer (Fig. 5a–c)These modes of interaction are in line with our experimental findings and explain why the tri-specific VHH-Fcs are more potent than the bi-specific or monoclonal VHH-Fcs.

## Discussion

In this study we developed and characterized llama-derived multi-specific nanobodies that yielded data that strongly suggests they will be effective against SARS-CoV-2 that causes COVID-19. The COVID-19 pandemic has caused widespread health and social issues around the globe, and requires therapeutics that can effectively stop and prevent the infection of SARS-CoV-2. Several monoclonal antibodies against SARS-CoV-2 have been suggested and being tested as anti-viral therapies, either as individual agents or combination therapies; however, this is the first study that introduces and demonstrates the efficacy of multi-specific antibodies against SARS-CoV-2 to our knowledge^10–15,19,30^.

To successfully design and construct multi-specific VHH binders, the epitope information for each individual VHH clone is necessary. Here, instead of obtaining crystal structures for each antigen/antibody complex, we utilized a different method for epitope identification. We initially performed epitope binning with biolayer interferometry and categorized S/ACE2 blocking VHHs into 2 groups. The VHHs within each group competed, but there was no competition with VHHs from the other group, strongly suggesting that Group 1 and Group 2 VHHs are two separate binding groups. Then, we computationally constructed VHH models and docked them separately to an S1 RBD structure obtained from a publicly-available crystal structure of SARS-COV-2 S1 RBD/ACE2, and utilized docking structures with higher pose cluster size to predict possible epitopes for the individual VHHs. To validate the involvement of predicted epitopes in VHH/S1 RBD binding, we compared the binding ability of each VHH to wild type S1 RBD and five deletion mutants with each predicted epitope deleted. As shown in Fig. 2, both Group 1 and Group 2 VHHs likely bind to the regions del3, del4, and del5 which overlap with the ACE2 binding interface of S1 RBD, however, at least part of the epitope for Group 2 is likely located more outwards of this region and has relatively less overlap with the ACE2 binding interface of S1 RBD. Currently, a number of structures of S1 RBD/antibody complexes have been published. The analysis of these structures show that there are likely 2 main “hot” antibody binding regions in S1 RBD: one likely in the N-terminal region (del1)^12,13^, and the other likely in the ACE2 binding interface (del3, del4, del5)^10,11,26^. Our selected VHH binders in tri-specific antibodies possibly cover both of these regions (Fig. 5). Based on this information, we were able to define the lead tri-specific VHH-Fc format, including the linker length and the order of the VHH binders.

The tri-specific antibodies are advantageous as therapeutic agents because they simultaneously bind multiple epitopes within the S1 protein RBD that increase their antigen-binding affinity and avidity (Fig. 5). The VHHs 1B and 3F that comprises the bi-specific antibody bind to two different epitopes in the S1 protein RBD^19^. In our tri-specific antibody design, we incorporated the VHH 2A that has an almost identical epitope as 1B. These VHHs could bind in different orientations to the same or similar epitopes, or to a corresponding epitope in another S1 protein in the trimer, increasing the binding and blocking potency of the tri-specific VHH-Fc. In fact, this phenomenon has been previously shown for other multi-specific antibodies. For example, the CD20 targeting T cell engager antibody CD20-TCB (Roche) with two CD20 binding domains (2:1 molecular format) has increased potency compared to other CD20-binding bi-specific antibodies in clinical development^31^. In agreement with this hypothesis, the resulting tri-specific VHH-Fcs showed very potent characteristics in terms of the S binding and S/ACE2 blocking efficacy, which are among the best in currently published anti-SARS-CoV-2 therapeutic antibodies (Table 1).

Because of these characteristics, the tri-specific VHH-Fcs could be used at low concentrations for therapeutic applications that would potentially lower their toxicity in humans. In addition, the strong binding of the antibodies to the virions would minimize the risk of antibody-dependent enhancement (ADE) that is caused by sub-optimal antigen–antibody interactions and promotes enhanced viral infections^32,33^. The multi-specific targeting approach also minimizes the loss of antibody binding to viral antigens due to the mutations of the viruses. The RNA viruses are known to mutate, and in this sense coronaviruses could lose the binding to antibodies relatively easily due to structural changes in the viral components^34,35^. However, the VHH multi-specific antibodies would still bind to the mutated virus since the other VHHs in the tri-specific antibody would bind the unmutated epitopes of the virus. Another advantage of the VHH multi-specific platform is the ability to target multiple viruses. For example, it is possible to adjoin VHHs that bind to other coronaviruses such as SARS-CoV and MERS-CoV, and construct pan-coronavirus tri-specific VHH-Fcs that would be effective in preventing and treating a broad spectrum of coronaviruses.

Our multi-specific antibody design connects human IgG1 Fc domain to bi- or tri-specific VHHs. Having the Fc domain in the antibody structure confers Fc-dependent cytotoxic functions such as ADCC, complement-dependent cytotoxicity (CDC) and antibody-dependent cellular phagocytosis (ADCP)^36–40^. These additional Fc-dependent functions, in addition to blocking virus entry and possible virus aggregation, would equip the VHH-Fcs with multiple mechanisms of action, making them more potent in neutralizing the coronaviruses. Indeed, our lead tri-specific VHH-Fc 3F-1B-2A show potent neutralization of SARS-CoV-2 pseudovirus infection in human cells.

One of the questions in the field of antibody therapeutics is whether the effect of using multi-specific single molecule is better than using a combination of monoclonal antibodies that collectively target the same epitopes or not^41^. Here, we show that multi-specific antibodies are more effective in blocking host-virus interactions than a combination of monoclonal antibodies. Our tri-specific VHH-Fc 3F-1B-2A was much more potent in blocking the SARS-CoV-2 S/ACE2 interaction than using VHH-Fcs 3F, 1B and 2A individually as a combination. It is likely that physically combining the VHHs increases overall association constants (K_on_ values) and decreases overall dissociation constants (K_off_ values), producing lower binding constants, thus increasing antibody affinity towards antigens. It also increases the avidity of the antibodies, making them more effective in neutralizing viruses.

One of the hallmarks of a successful therapeutic antibody is its developability features^42,43^. Especially during pandemics such as COVID-19 when rapid production of antibodies in high quantities is essential, the developability and manufacturability of the antibodies play even crucial roles. Our design has the advantage of using llama VHH nanobodies that have high stability^17,18^. Indeed, the biochemical and biophysical characteristics of the multi-specific VHH-Fc show that they can be purified in high quantity, have better aggregation resistance, and have favorable thermostability. In addition, our antibodies have high developability because the multi-specific design combines the individual VHHs into single molecules instead of combinations, making their manufacturing easier. An alternative strategy of increasing developability of the anti-SARS-CoV-2 multi-specific antibodies would be to combine 4 VHHs without the addition of IgG Fc domain to construct tetra-specific VHHs. These molecules would have the added advantage of increased affinity and avidity towards SARS-CoV-2 S1 protein compared to bi- and tri-specific VHH-Fcs, despite lacking the Fc effector functions. These tetra-specific antibodies would be ideally suited as antibody prophylactic to prevent the SARS-CoV-2 infection in humans because their llama VHH-only structure would have increased thermostability, easier combination capability, and the possibility of easy large-scale manufacturing using cost-effective expression systems such as Yeast^17^.

One of the key features of our therapeutic antibodies is the use of computer-aided design that greatly reduces their development time and enhances their optimization efficiency. For instance, from the inception of this project, it was possible to produce, optimize and test our lead tri-specific VHH-Fcs in less than 3 months. This shows that this strategy is powerful for producing novel therapeutic antibodies for time-sensitive unmet needs, and can be utilized for future outbreaks that would require rapid development of antibody therapeutics.

## Materials and methods

### Cell lines and transfections

The cell lines used in this study were cultured in media as stated below. Expi293 (Thermo Fisher Scientific)—Expi293 expression medium (Thermo Fisher Scientific), NK-92-CD16 (Natural killer cell line expressing CD16) cells (ATCC)—RPMI 1640, 10% fetal calf serum (FCS), 40 ng/ml IL-2. The cells were maintained in a humidified chamber at 37 °C, 8% CO_2_. The Expi293 cells were transiently transfected with plasmids expressing SARS-CoV-2 S1 protein using the ExpiFectamine 293 transfection reagent (Thermo Fisher Scientific) according to manufacturer’s instructions. Briefly, the cells were plated at 1.7 × 10^6^ cells/ml density overnight in 30 ml of fresh media in 125 ml shake flasks. The following day, 30 μg of DNA and 80 µl of ExpiFectamine 293 were separately mixed in 1.5 ml of Opti-MEM, and incubated at room temperature for 3 min. Then, the ExpiFectamine 293 and DNA mixtures were mixed and incubated for another 20 min at room temperature. Finally, it was added to flasks containing the cells and incubated in a humidified chamber at 37 °C, 8% CO_2_. The cells were used for experiments after overnight incubation or frozen in liquid N_2_.

### Generation of deletion mutants of the SARS-CoV-2 S protein

The codon-optimized version of the open reading frame cDNA for SARS-CoV-2 S protein was purchased from Sino Biological in the vector pCMV3-SP-N-Myc. The deletion mutants of the SARS-CoV-2 S were generated by the QuikChange II XL Site-Directed Mutagenesis Kit (Agilent Technologies, Cat.200522) according to manufacturer’s instructions. The primer sequences used are listed in the Supplementary Table 1.

### VHH-Fc expression and purification

The Expi293 cells were transiently transfected with plasmids expressing VHH-Fcs as stated previously according to manufacturer’s instructions. Enhancers were added to cells 17 h after transfection and they were centrifuged at 3000*g* for 10 min after 72 h of transfection. Then, the supernatant was filtered with a 0.45 µm membrane and the antibody concentration was determined using Protein A probe on Gator (Probe Life). Then, the VHH-Fcs were purified using Protein A columns on the AKTA Explorer 100 purification system (buffer A: PBS, pH = 7.4; buffer B: 0.1 M Glycine, pH = 2.5), and dialyzed in PBS twice. The antibodies were then filtered again with a 0.22 µM membrane and used for experiments.

### Epitope binning (competition) assays

The initial assay was performed using Gator system (Probe Life). After pre-wetting the SARS-CoV-2 S1 RBD sensors in Q Buffer (Probe Life), the sensor captured 10–30 µg/ml of the first monoclonal VHH-Fc for about 300 s, then the loaded sensor captured 10 µg/ml of the second monoclonal VHH-Fc, either 1B, 3F, or 2A, which was quantified over time by Gator.

The follow-up assay for VHH-Fcs 1B-2A and 3F was performed by ELISA. A 96-well plate was coated to a final concentration of 1 µg/ml of SARS-CoV-2 S1 protein and placed overnight at 4ºC. To test the binding with VHHs 1C, 1F, 3A, 4F and G4, the following method was used. 1B-2A-Fc or 3F-Fc at 60 µg/ml were premixed with each competing c-Myc tagged VHHs from periplasmic supernatant at a 1:1 ratio. After another one hour of incubation, a biotinylated anti-c-Myc antibody (9E10) was added and the samples were incubated for another one hour. Then, streptavidin-HRP was added followed by the treatment with Amplex Red (Thermo Fisher Scientific) and 30% H_2_O_2_ containing development solution. The emitted signal for each sample was detected by using a fluorescence plate reader (SpectraMax Gemini XPS). To test the binding with VHHs 1B-2A-Fc and 3F-Fc, the following method was used. 1B-2A-Fc or 3F-Fc at 50 µg/ml were premixed with competing biotinylated 1B-2A-Fc or 3F-Fc at a 10:1 ratio. After one hour of incubation, the biotin-streptavidin detection system as described above was used to analyze their competition. The percent difference from the competing pairs versus the VHH-Fc alone signal was calculated using the following formula; % difference from VHH-Fc signal = (1 − ((signal of competing pair − no antibody signal)/(signal of VHH-Fc alone − no antibody signal)) × 100.

### Cell binding assay

Binding of VHH-Fcs to SARS-CoV-2 S1 expressing cells was assessed by flow cytometry. Briefly, cells were harvested and resuspended in PBS with 2% fetal bovine serum (FBS) and plated in v-bottom 96-well plates at 5 × 10^4^ cells/well density. They were incubated for 1 h at room temperature with 10 µg/ml of indicated VHH-Fcs, control antibodies or recombinant biotinylated human ACE2 also dissolved in PBS with 2% FBS. Then, the cells were washed twice with the same buffer, and incubated with FITC-conjugated Goat anti-human IgG (Jackson ImmunoResearch) at 1:200 dilution or PE-conjugated streptavidin (Thermo Fisher, for the detection of biotinylated ACE2) at 1:500 dilution for 30 min at room temperature. Cells were washed again following the secondary antibody treatment. Then they were analyzed by a FACSCalibur cytometer (BD Biosciences). Cell populations were visualized as forward vs side scatter and gated to exclude dead cells. Cells treated with no antibodies were used to establish background fluorescence. The resulting FACS data were analyzed by the software FlowJo (BD Biosciences) and the graphs were generated by the software Prism (GraphPad).

### In-vitro S1 protein binding assay

The 96-well ELISA plates (Greiner Bio-One) were directly coated with SARS-CoV-2 S1 protein (Acro Biosystems) diluted in PBS at 1 µg/ml and incubated overnight at 4 °C. Then, the plates were washed with PBS containing 0.5% Tween20 (PBST) and blocked with 1% BSA in PBS at room temperature for one hour. The plates were washed again with PBST and incubated with the test antibodies at room temperature for one hour. The antibodies were used at 1:5 serial dilutions. The plates were washed with PBST followed by the addition of anti-human-Fc antibodies conjugated to horseradish peroxidase (HRP) (Jackson ImmunoResearch) at 1:5000 dilution in PBST and the plates were incubated at room temperature for 1 h. Following washing again by PBST, the plates were treated with ELISA development buffer solution containing Amplex Red and 30% H_2_O_2_. The emitted binding signal for each sample was detected by using a fluorescence plate reader. The blocking was measured in relative fluorescence units (RFU) and the % inhibition was calculated as follows; % Inhibition = (1 − (mean experimental value/mean of no antibody control)) × 100.

### S/ACE2 blocking assay

The 96-well ELISA plates (Greiner Bio-One) were coated with SARS-CoV-2 S1 protein (Acro Biosystems) and incubated overnight as stated previously. Then, the plates were washed with PBST and blocked with 2% BSA in PBS at room temperature for one hour. The plates were washed again with PBST and incubated with the test antibodies at room temperature for 45 min. The antibodies were used at 1:5 serial dilutions. Then, recombinant biotinylated-ACE2 (Acro Biosystems) was directly added to the plates at 4.65 µg/µl and incubated at room temperature for another 45 min. The plates were washed with PBST followed by the addition of streptavidin conjugated to HRP at 1:1000 dilution in PBST. The plates were incubated at room temperature for another 45 min. Then, they were washed with PBST and treated with ELISA development buffer. The emitted binding signal for each sample was detected by using a fluorescence plate reader.

### Analysis of physical characteristics of VHH-Fcs

The purified VHH-Fcs were analyzed by the UNcle system (Unchained Labs) for their thermostability using differential scanning fluorimetry (DSF) and static light scattering (SLS), and aggregation potential using dynamic light scattering (DLS) assays. The DLS was measured at 25 °C and the data was analyzed using UNcle Analysis Software. For DSF/DLS assays, a temperature ramp of 1 °C/min was performed with monitoring from 25 to 95 °C. SLS was measured by UNcle at 266 nm and 473 nm. Tm and Tagg were analyzed and calculated by the UNcle Analysis Software.

### Pseudovirus neutralization assay

Pseudovirus neutralization assay was performed in collaboration with GenScript Biotech (Piscataway, NJ). Briefly, pseudovirus expressing luciferase and containing SARS-CoV-2 S1 as the envelope glycoprotein in a lentiviral vector was produced in HEK293T cells, and the virus titration was determined by ELISA. HEK293 cells expressing ACE2 receptor and transmembrane Serine protease 2 (TMPRSS2) were used as the target cells, and were seeded in 96-well plates. Then, pseudovirus with the serial dilutions of the antibodies were mixed with the target cells. The cells were incubated for 48 h at 37 °C and an amount of 30 µl of the cell suspension was transferred to an assay plate. It was mixed with luciferase detection reagents from Bio-Glo™ Luciferase Assay System (Promega) and incubated for 5–10 min at room temperature. Then, the luminescence was measured by a plate reader. The background RLU was subtracted from the RLU of the experimental samples. The values for % inhibition were derived from RLU as follows; % Inhibition = (1 − (mean of experimental value − mean of cells treated only with buffer)/(mean of cells treated only with SARS-CoV-2)) × 100.

### Antibody-dependent cellular cytotoxicity (ADCC) assay

Target Expi293 cells expressing S1 protein (293 SProt) were washed with RPMI media containing 10% Horse serum and 40 ng/ml IL-2, and plated in 96-well plates at 1 × 10^4^ cells/well density. Then, they were mixed with antibodies at 40 µg/ml of final concentration. Then, NK-92-CD16 cells expressing GFP were added to wells at 3 × 10^4^ cells/well density (Effector: Target—3:1) and the plates were incubated overnight 37 °C, 8% CO_2_. Then, the cells were washed twice and resuspended in DPBS with 2% FBS. They were assessed by flow cytometry using a FACSCalibur cytometer (BD Biosciences). 293 SProt and GFP-NK-92-CD16 cells were each used as a reference to set up overall target cell gating and to establish the GFP positive NK-92-CD16 populations, allowing differentiation between the NK-92-CD16 effector cells and 293 SProt target cells. The GFP negative 293 SProt cell percentage was evaluated for all samples. Then, cell death percentage was calculated as follows; % Cell death = (1 − (antibody treated cell percentage/average of isotype control percentage)) × 100.

### Statistical analysis

The four-parameter non-linear regression analysis from Prism software version 8.4.3 was used for all binding and blocking curves, which also included the IC_50_ values for the blocking assays. All error bars represented in the data are based on standard deviation, unless otherwise specified.

## Supplementary information

Supplementary Information.

## Data Availability

The data generated and/or analyzed during this study are available from the corresponding author on reasonable request.
