# Author Correction: Development of humanized tri-specific nanobodies with potent neutralization for SARS-CoV-2

**DOI:** 10.1038/s41598-021-82913-x

**Published:** 2021-02-17

**Authors:** Jianbo Dong, Betty Huang, Bo Wang, Allison Titong, Sachith Gallolu Kankanamalage, Zhejun Jia, Meredith Wright, Pannaga Parthasarathy, Yue Liu

**Affiliations:** 1 Ab Studio Inc., Hayward, CA USA; 2 Ab Therapeutics Inc., Hayward, CA USA

Correction to: *Scientific Reports* https://doi.org/10.1038/s41598-020-74761-y, published online 20 October 2020

In the original version of this Article, Jianbo Dong and Betty Huang were omitted as equally contributing authors. This has now been corrected in the PDF and HTML versions of the paper.

