# Supplementary material for: Development of humanized tri-specific nanobodies with potent neutralization for SARS-CoV-2
Source: Sci Rep. 2020 Oct 20;10:17806. doi: 10.1038/s41598-020-74761-y (PMC7576208; doi:10.1038/s41598-020-74761-y)
Supplement: Supplementary file 1 — Supplementary Information. [file 41598_2020_74761_MOESM1_ESM.pdf]

Development of humanized tri-specific nanobodies with potent neutralization for SARS-CoV-2

Jianbo Dong[1,*], Betty Huang[1], Bo Wang[1], Allison Titong[1], Sachith Gallolu Kankanamalage[1], Zhejun Jia[1], Meredith Wright[1], Pannaga Parthasarathy[1] and Yue Liu[1,2]

1. Ab Studio Inc., Hayward, CA, USA

2. Ab Therapeutics Inc., Hayward, CA, USA

* Corresponding author (jianbo.dong@antibodystudio.com)

Supplementary Information

Supplementary Tables

Supplementary Table 1: Primer sequences used to generate SARS-CoV-2 S1 RBD deletion mutants

deletion mutant	Forward primer	Reverse primer
del1	ccaacctgtgtccatttggaagcaactgtgtggc	gccacacagttgcttccaaatggacacaggttgg
del2	ctatgctgactcctttgtgtacaaactgcctgatgact	agtcatcaggcagtttgtacacaaaggagtcagcatag
del3	gcaaggtgggaggctaccaggctggcag	ctgccagcctggtagcctcccaccttgc
del4	ggttggttggaagccatatccattacatggtgtgct	agcacaccatgtaatggatatggcttccaaccaacc
del5	ccaatcctatggcttccaaccatacagggtgg	ccaccctgtatggttggaagccataggattgg

Supplementary Figures

Supplementary Figure 1

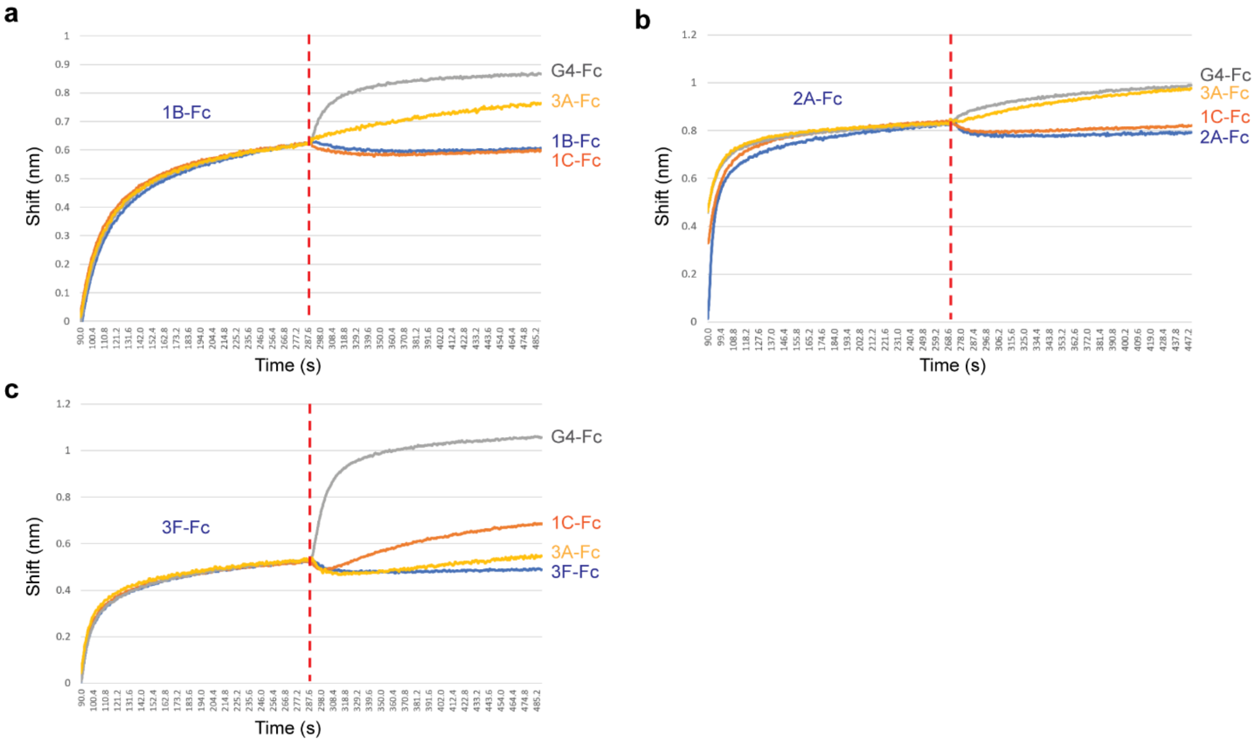

<u>Supplementary Figure 1: Epitope binning assay to validate the competition among S1 RBD binding VHH-Fcs</u>

The VHH-Fcs 1C, G4, and 3A that were initially used to assess binding competition by ELISA (Figure 1d) were assessed by biolayer interferometry using Gator (Probe Life) for their binding competition for S1 RBD. **a)** 1B-Fc-loaded, **b)** 2A-Fc-loaded, and **c)** 3F-Fc-loaded RBD sensors were used to capture indicated VHH-Fcs and the resulting quantitation of the wavelength shift that corresponds to the binding signal over time are shown in the graphs.

Supplementary Figure 2

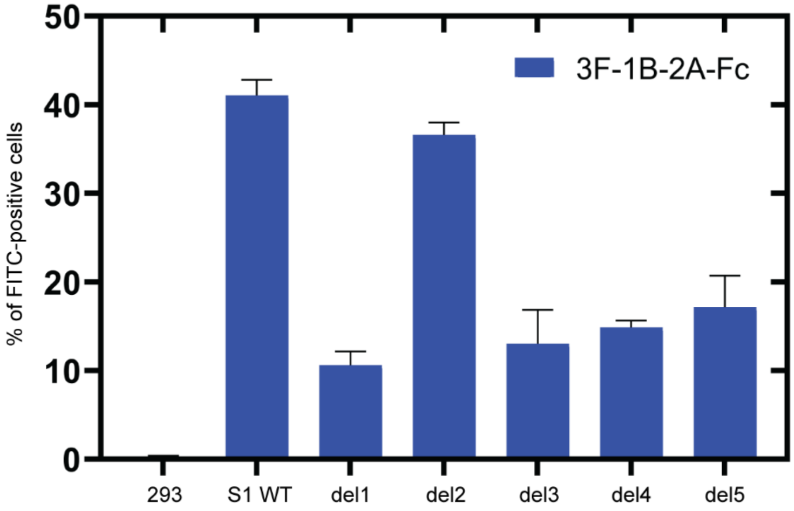

Supplementary Figure 2: Assessment of folding of SARS-CoV-2 S1 RBD wild type and deletion mutants

The binding of tri-specific VHH-Fc 3F-1B-2A to Expi293 cells expressing wild-type SARS-CoV-2 S1 or deletion mutants (del1-del5) were analyzed by flow cytometry following FITC-conjugated secondary antibody treatment. The experiment was performed at least three times with similar trends in results. The experiment shown here was performed in triplicates and the error bars represent standard deviation.

Supplementary Figure 3

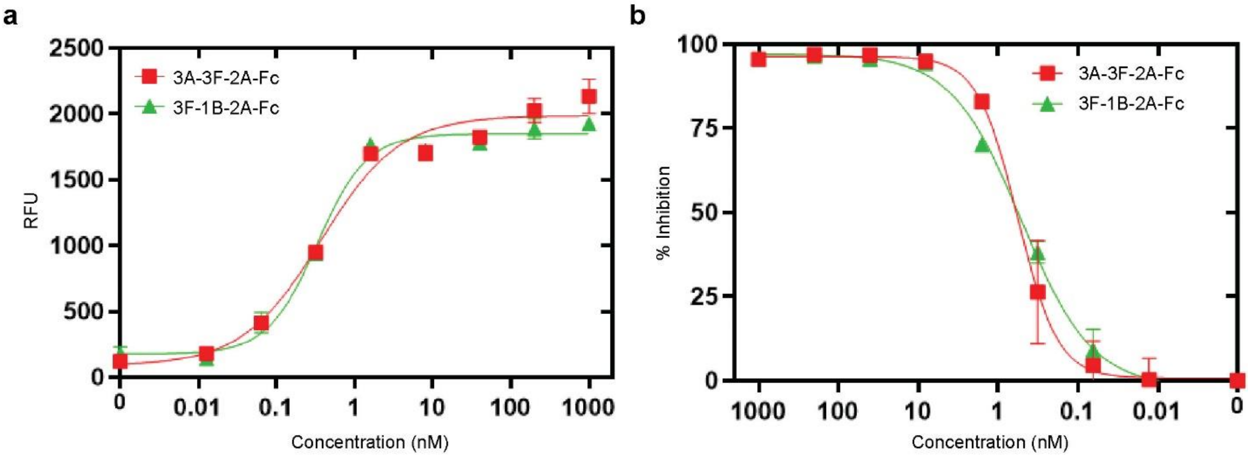

Supplementary Figure 3: SARS-CoV-2 S1 RBD binding and S/ACE2 blocking by tri-specific VHH-Fc 3A-3F-2A

a) Binding of tri-specific VHH-Fcs 3A-3F-2A and 3F-1B-2A to SARS-CoV-2 S1 protein at different concentrations in duplicates using an ELISA method. The binding signal is based on fluorescence, indicated as Relative Fluorescence Units (RFU). Error bars represent standard deviation. **b**) Blocking of SARS-CoV-2 S/ACE2 interaction by VHH-Fcs 3A-3F-2A and 3F-1B-2A at different concentrations in duplicates using an ELISA method. Percent inhibition was calculated based on the blocking signal in RFU for each VHH-Fc treatment. Error bars represent standard deviation.
